# Identification of a Predominant Co-Regulation among Kinetochore Genes, Prospective Regulatory Elements, and Association with Genomic Instability

**DOI:** 10.1371/journal.pone.0025991

**Published:** 2011-10-10

**Authors:** William C. Reinhold, Indri Erliandri, Hongfang Liu, Gabriele Zoppoli, Yves Pommier, Vladimir Larionov

**Affiliations:** 1 Laboratory of Molecular Pharmacology, Center for Cancer Research, National Cancer Institute, National Institutes of Health, Bethesda, Maryland, United States of America; 2 Division of Biomedical Statistics and Informatics, Mayo Clinic, Rochester, Minnesota, United States of America; 3 Department of Internal Medicine, University of Genova, Genova, Italy; Niels Bohr Institute, Denmark

## Abstract

The NCI-60 cell line panel is the most extensively characterized set of cells in existence, and has been used extensively as a screening tool for drug discovery. Previously, the potential of this panel has not been applied to the fundamental cellular processes of chromosome segregation. In the current study, we used data from multiple microarray platforms accumulated for the NCI-60 to characterize an expression pattern of genes involved in kinetochore assembly. This analysis revealed that 17 genes encoding the constitutive centromere associated network of the kinetochore core (the CCAN complex) plus four additional genes with established importance in kinetochore maintenance (CENPE, CENPF, INCENP, and MIS12) exhibit similar patterns of expression in the NCI-60, suggesting a mechanism for co-regulated transcription of these genes which is maintained despite the multiple genetic and epigenetic rearrangements accumulated in these cells (such as variations in DNA copy number and karyotypic complexity). A complex group of potential regulatory influences are identified for these genes, including the transcription factors CREB1, E2F1, FOXE1, and FOXM1, DNA copy number variation, and microRNAs has-miR-200a, 23a, 23b, 30a, 30c, 27b, 374b, 365. Thus, our results provide a template for experimental studies on the regulation of genes encoding kinetochore proteins, the process that, when aberrant, leads to the aneuploidy that is a hallmark of many cancers. We propose that the comparison of expression profiles in the NCI-60 cell line panel could be a tool for the identification of other gene groups whose products are involved in the assembly of organelle protein complexes.

## Introduction

Chromosome segregation in eukaryotes requires a multi-protein structure termed the kinetochore, which assembles on centromeric DNA to mediate both the binding of spindle microtubules to chromosomes and chromosome movement. Despite the great divergence of centromeric DNA sequences among vertebrates, kinetochore structure and composition is highly conserved. The kinetochore in vertebrates appears as trilaminar plates, with electron dense inner and outer plates, and an electron lucent middle layer ([1] and references therein). The inner kinetochore that is apposed to centromeric DNA is essential for kinetochore assembly. In particular, the centromere-specific histone H3 variant CENPA localizes in the inner plate and functions in the early organization of centromeric chromatin structure during interphase [2], [3]. CENPA is a key element of eukaryotic centromeres. Other kinetochore proteins interact with CENPA-containing nucleosomes, leading to the assembly of a functional kinetochore. Currently, about 90 kinetochore proteins have been identified in humans [4], [5], [6]. The proteins of this complex are recruited to the kinetochore at different stages of mitosis.

The kinetochore has a dynamic organization and most of the proteins are recruited to it during late G2 phase, and are then either depleted following microtubule attachment or persist until the onset of anaphase or the end of mitosis [7], [8]. Purification of CENPA nucleosomes from human cells identified a set of proteins that are constitutively present at centromeres, the constitutive centromere associated network, or CCAN. The CCAN network is comprised of 17 interacting proteins, CENPA, CENPB, CENPC (CENPC1), CENPS (APITD1), CENPW (C6orf173), CENPH, CENPI, CENPK, CENPL, CENPM, CENPN, CENPO, CENPP, CENPQ, CENPT, CENPR (ITGB3BP), and CENPU (MLF1IP) [9], [10], [11], [12].

Besides those proteins included in the CCAN, there are several other proteins that localize to the centromere throughout the cell cycle. Included are MIS12, CENPE, CENPF and INCENP, kinetochore proteins that have been shown to have a fundamental role in kinetochore formation [5], [12], [13], [14], [15]. The highly conserved protein MIS12 forms a complex with both the heterochromatin proteins and the outer kinetochore proteins [9], [16]. Thus, MIS12 is a bridge that connects the inner and outer kinetochore. Its depletion results in chromosomal mis-segregation and loss of CENPA, CENPH and CENPE [15]. CENPE and CENPF are involved in microtubule capture, spindle checkpoint modulation, and kinetochore-microtubule interface stability [17], [18]. A chromosome lacking CENPE is unable to congregate along the nuclear equator during mitosis [18]. Likewise, CENPF knock-out cells suffer from microtubule dysfunction [17]. The incorrect microtubule attachment that leads to chromosome mis-segregation can be repaired by the chromosomal passenger complex (CPC), which includes the inner centromere protein INCENP [14], [19].

There are several publications reporting that transient depletions or over-expressions of one of the proteins involved in kinetochore complex formation lead to aneuploidy and polyploidy, hallmarks of many cancers [20], [21], [22], [23], [24]. Thus, kinetochore assembly represents a well-coordinated process requiring synthesis of a stochiometric amount of kinetochore proteins in the cell. However, currently no information is available on regulation of kinetochore-associated genes.

In this study, we explored the National Cancer Institute 60 cell line panel (NCI-60), derived from nine tissue–of-origin types of cancer, to analyze the pattern of expression for 21 kinetochore associated genes [25]. The NCI-60 were selected and developed by the Developmental Therapeutics Program at the NCI to act as a screen for the potential efficacy of compounds for use as anti-cancer agents. To this end, many thousands of compounds have been tested for growth inhibition on this screen. In addition, the NCI-60 cell lines have been characterized in multiple additional manners, including transcript expression, proteomic profiling, bacterial artificial chromosome microarrays-based DNA copy number determinations, and microRNA expression levels [26], [27], [28], [29], and their genetic identities have been fingerprinted excluding possible cross-contamination [30]. Our analysis first identified a predominant pattern of co-regulation among the 21 genes known to be present in the kinetochore core during the cell cycle. Several regulatory elements with significant correlation to the genes expression levels were identified in promoter regions of kinetochore associated genes, including the transcriptional regulators CREB1, E2F1, FOXE1, and FOXM1, and several microRNAs, implying a multi-factorial transcriptional regulation for the genes. In addition, transcript expression level fluctuations were found to be associated with karyotypic instability.

## Materials and Methods

### Transcript probe set and probe data

Transcript expression for each gene was determined starting with all pertinent probes from five platforms. From Affymetrix (Affymetrix Inc., Sunnyvale, CA) we used the Human Genome U95 Set (HG-U95) with ∼60,000 features [31], [32]; the Human Genome U133 (HG-U133) with ∼44,000 features [31], [32] (Gene Expression Omnibus, GEO, accession number GSE5949); the Human Genome U133 Plus 2.0 Arrays (HG-U133 Plus 2.0) with ∼47,000 features [32] (GEO accession number GPL570); and the GeneChip Human Exon 1.0 ST array (GH Exon 1.0 ST) with ∼5,500,000 features [33] (GEO accession number GSE29682). From Agilent (Agilent Technologies, Inc., Santa Clara, CA) we used the Whole Human Genome Oligo Microarray, with ∼41,000 features [29], [32] (GEO accession number GSE22821). HG-U95 and HG-U133 were normalized by GCRMA [34]. HG-U133 Plus 2.0 and the Whole Human Genome Oligo Microarray were normalized by RMA [35]. All Agilent mRNA probes considered to be detected in at least 10% of the cell lines were normalized using GeneSpring GX by i) setting any gProcessedSignal value less than 5 to 5, ii) transforming the gProcessedSignal or gTotalGeneSignal to Logbase 2, and iii) normalizing per array to the 75^th^ percentile [29]. All transcript microarrays were done using materials generated by the Genomics and Bioinformatics Group (GBG), as well as being carried out by the GBG and its collaborators.

**Figure 1:**
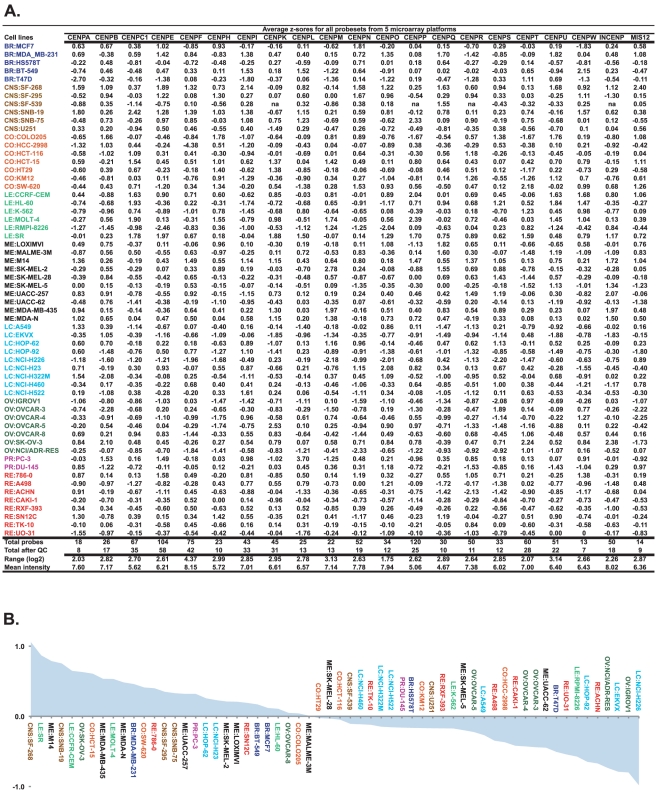
Transcript levels for 21 kinetochore genes in the NCI-60. A. Average z score values calculated from multiple probes yielding relative transcript expression levels. Average z scores were calculated from each group of probeset intensities for the NCI-60, and then averaged by cell line. “QC” in the third row from the bottom is “quality control”. For the calculations of “range”, in the second row from the bottom, minimum and maximum values are first calculated across the NCI-60 for each probeset for a gene. The maximum minus the minimum is the range for that probeset. The average of the probeset ranges is the composite range shown here. For the calculations of “mean intensity” in the bottom row, log_2_ average intensity is first calculated for the NCI-60 for each probeset for a gene. The average of these log2 values is then taken to give the composite mean intensity shown here. B. Average z scores calculated for each cell line from the 21 kinetochore gene values (from each row of Figure 1A), in descending order. The x-axis is the 60 cell lines in the NCI-60. For both A and B, the cell lines are color coded by tissue of origin type. The y-axis is the average z score.

Inclusion of probes (Agilent) or probe sets (Affymetrix) in the determination of relative gene expression levels was dependent on their passing quality control criteria, done as follows. Average probe set (meant to include Agilent probes in the following text) intensity ranges were determined, and all with an intensity range < or equal to 1.2 log_2_ were dropped. The number of probe sets that passed this criteria for each gene was determined, and 25% of that number calculated. For the remaining probe sets for each gene, Pearson's correlations were determined for all possible combinations. The average correlation for each probe set was determined as compared to all others for each gene. All probe sets whose average correlations were less than 0.30 were dropped. Next, if there were probe sets with average correlations less than 0.60, we dropped the probe set with the lowest correlation. Correlations were recalculated for the remaining possible probe set/probe set combinations. Probe sets with the lowest average correlations continued to be dropped, and the average recalculated until either all average correlations were ≥ to 0.60, or the 25% level of the original probe set number (calculated above) was reached. Of the 21 known kinetochore genes included in this study (Figure 1A and B), one (CENPR) reached that 25% threshold criteria.

### Z score determinations

In order to obtain a single composite value of the probe and probe set intensities that passed quality controls criteria, intensities were transformed into z scores [36], by subtracting their 60 cell line means, and dividing by their standard deviations. Average z scores were determined for all available (16,820) genes across all probes and probe sets for each cell line (see Figure 1A). These calculations were done in Java.

### Kinetochore transcript expression correlation and clustering

**Figure 2:**
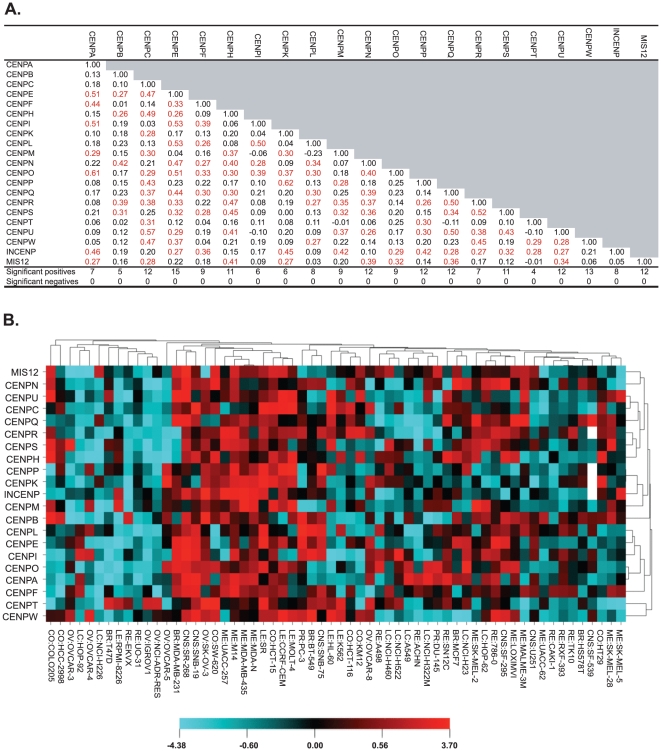
Kinetochore transcript expression correlation and clustering. A. Pearson's correlations between the transcript expression level patterns of 21 known kinetochore genes (Figure 1A). Statistically significant correlations at p<0.05 (without multiple comparisons correction) are red. In the last two rows, the “Significant positives” and “Significant negatives” are the number of statistically significant positive, or negative correlations for that gene as compared to the 20 other kinetochore genes. B. Cluster image map of the relative transcript expression levels for the kinetochore genes (from Figure 1A) in the NCI-60. The cell lines are plotted on the x-axis. The kinetochore genes are plotted on the y-axis. Both axes were clustered based on Euclidean distance, with average linkage.

The correlations in Figure 2A are Pearson's, and were calculated using Excel 2008 for Mac. The cluster image map in Figure 1B was generated using CIMminer (http://discover.nci.nih.gov/cimminer/).

### Distribution of correlation analysis

**Figure 3:**
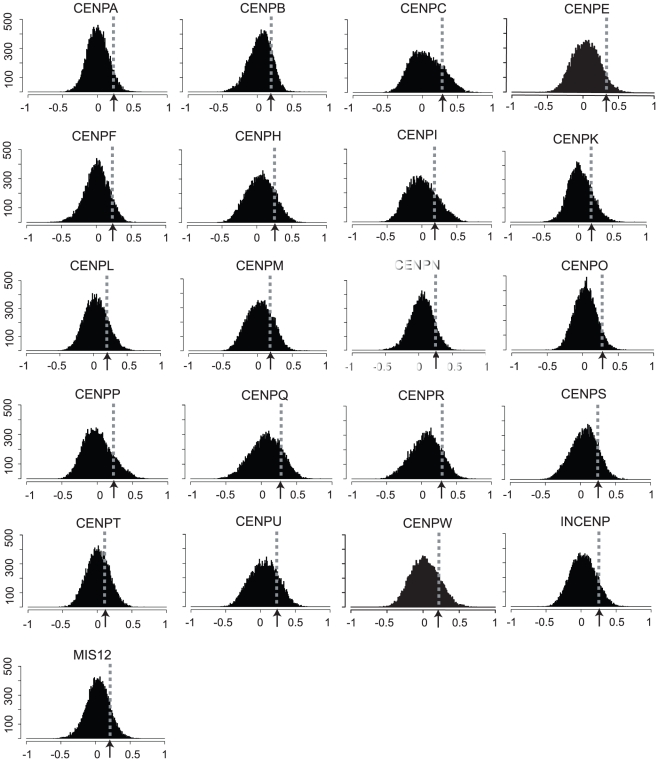
Distribution of Pearson's correlations of the transcript expression levels for 21 known kinetochore genes versus all other available genes. The average z score for each of 16,820 genes was calculated as for the known kinetochore genes (Figure 1A) for the NCI-60. The correlation values are plotted on the x-axis. The frequency of genes at each level of correlation is plotted on the y-axis.

The distribution pattern of the kinetochore gene z scores' (from Figure 1A) correlated to all other genes z scores shown in Figure 3 were calculated using R (http://www.r-project.org/).

### Regulatory factor analysis

**Figure 4:**
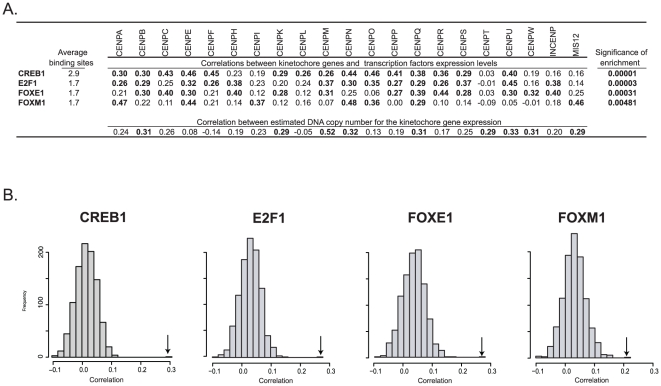
Association between the expression levels of 21 kinetochore genes (**Table 1**), to transcription factors expression and DNA copy number. A. The average number of transcription factor binding sites per kinetochore gene is presented in the “Average binding sites” column. The “Correlation between kinetochore genes and transcription factors expression levels” columns presents the Pearson's correlations between each transcription factor/kinetochore pairing, with statistically significant correlations (p<0.05, without multiple comparisons correction) in bold. The “significance of enrichment” column depicts p values for level of enrichment of the average correlation of the transcription factor to the kinetochore genes, as compared to all genes computed using 1,000 random samples of 21 genes. The “Correlation between estimated DNA copy number for the kinetochore gene expression” calculations were done using the kinetochore gene expression values from Figure 1A, and DNA copy numbers determined from NimbleGen Systems Inc. HG18 CGH 385K WG Tiling v2.0 arrays. B. The distribution of correlations of transcription factor expression to all other genes, computed using 1,000 random samples of 21 genes. Correlation values are plotted on the x-axis. The frequency of 21 gene groups at each level of correlation is plotted on the y-axis. The mean correlation between the transcription factor and the kinetochore genes is indicated by the arrow.

The average number of transcription factor binding sites per kinetochore gene in Figure 4A were determined using data from the ABCC GRID Promoter Feature Extraction Page at http://grid.abcc.ncifcrf.gov/promoters/promoterInfo.php. Several of the gene designations were non-specific, including CREB, E2F, and FOX, so multiple family members were checked. Correlations between transcription factors and kinetochore genes in Figure 4A were Pearson's, and were based on transcription factor expression levels (data not shown), calculated as described for the kinetochore genes (see Figure 1A). Significance of enrichment calculations were made using R (http://www.r-project.org/).

The correlations between kinetochore gene expression (from Figure 1A) and DNA copy number done in Figure 4A were based on intensity values used for estimation of DNA copy number were from NimbleGen Systems Inc. HG18 CGH 385K WG Tiling v2.0 array. Data from this array can be accessed at our relational database, CellMiner, at http://discover.nci.nih.gov.

Probes specific for each of the 21 kinetochore genes (Figure 1A) plus seven flanking p and q terminal probes were used to estimate DNA copy numbers. The estimated copy number was calculated as
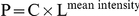
for which C = 2 (the correction for generating the intensities as a ratio of the cell line intensity to a normal, 2N, DNA), and L = 2 (the log of the intensity values).

All Figure 4 correlations are Pearson's, and were calculated in Excel 2008 for Mac. The correlation distribution graphs in Figure 4B were generated using R (http://www.r-project.org/).

### microRNA expression level determination

The purification, quality assessment, and expression level determinations of the microRNAs has been described previously [29]. In brief, 100 ng of total RNA was labeled as recommended by Agilent Technologies (miRNA Microarray System Protocol v 1.5). Labeled samples were hybridized to the Agilent Technologies Human miRNA Microarray (V2). Arrays were scanned and the data extracted as recommended by Agilent Technologies. The microRNA expression data is available at http://discover.nci.nih.gov/cellminer/. The correlations in Table 1 are Pearson's, and were calculated in Excel 2008 for Mac. The five-microarray z scores for the 16,820 available genes were used in this analysis.

**Table 1:** Kinetochore genes with both significant correlation to microRNA expression levels, and microRNA binding sites.^a^

	miRNAs^a^	
		Correlation to kinetochore
Gene	Identifier	gene expression
CENPA	hsa-miR-200a	**−0.26**
CENPC	hsa-miR-23a	**−0.32**
	hsa-miR-23b	**−0.31**
CENPH	hsa-miR-30a	**−0.53**
	hsa-miR-30c	**−0.28**
CENPK	hsa-miR-27b	**−0.28**
	hsa-miR-374b	**−0.27**
CENPR	has-miR-365	**−0.36**
CENPU	hsa-miR-23a	**−0.31**
	hsa-miR-27a	**−0.29**
	hsa-miR-30a	**−0.35**

a Only gene miRNA combinations with both significant negative homology, as well as 3′ miRNA binding sites are listed.

### Functional categorization

**Figure 5:**
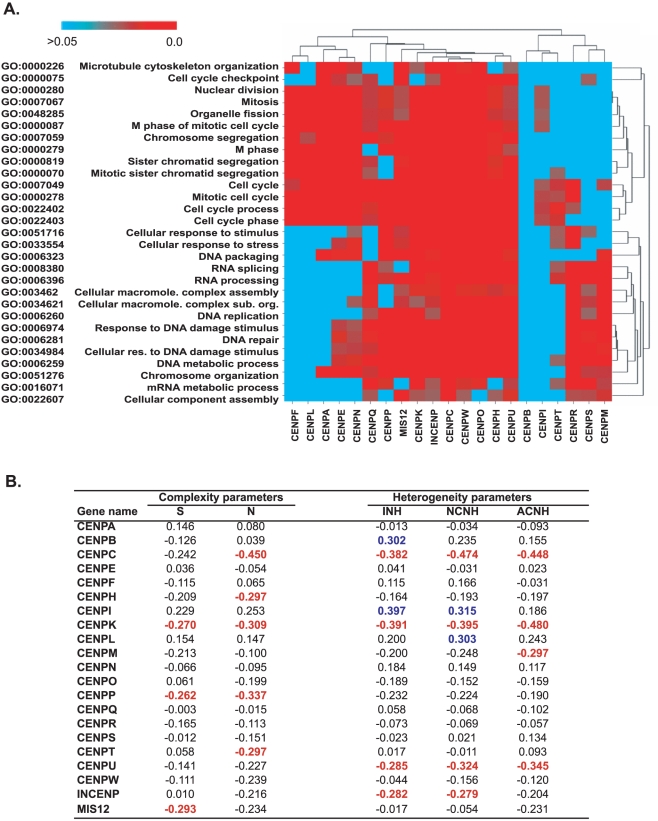
Significantly altered functional categories for those genes with significant correlation to kinetochore genes, and association of kinetochore gene expression with genomic instability. A. Identification of enriched functional categories in those 21 groups of genes correlated to the kinetochore genes at statistically significant levels (p<0.05) by expression pattern. The x-axis is the 21 kinetochore groups of genes with significant correlation to kinetochore genes. The y-axis is 29 GO functional categories with significant enrichment for at least 11 kinetochore gene groups. The color bar defines the false discovery rate, with the reds indicating the significantly enriched groups. Both axes were clustered based on Euclidean distance, with average linkage. B. Pearson's correlations between parameters of chromosomal instability [37] and kinetochore gene transcript levels (Figure 1A) for the NCI-60. **S** is the number of clonal **s**tructurally rearranged chromosomes. **N** is the **n**umerical complexity, ie the number of whole chromosome number gains and losses, as compared to the cell line ploidy level. **INH** is the **i**ndex of **n**umerical **h**eterogeneity. This is a summation of the number of centromeres with gains (in 2 or more cells) or losses (in 3 or more cells). **NCNH** is the fraction of **n**ormal **c**hromosomes that experience **n**umerical **h**eterogeneity. These are the gains or losses of normal chromosomes with the same centromeres. **ACNH** is the fraction of **a**bnormal **c**hromosomes that experience **n**umerical **h**eterogeneity. These are the gains or losses of abnormal chromosomes with the same centromeres. Bold red and blue type indicates negative or positive statistical significance (without multiple comparisons correction) at p<0.05, respectively.

Genes that were correlated to kinetochore gene expression patterns (Figure 1A) at statistically significant levels (p<0.05) were determined, and then assessed for significant enrichment of functional categories based on the Gene Ontology (http://www.geneontology.org/) and using High-Throughput GoMiner (http://discover.nci.nih.gov/gominer/htgm.jsp) for category identification. Those functional categories with significant change (p<0.05) in at least 11 of the 21 kinetochore genes are presented in Figure 5A. The cluster image map was generated using CIMminer (http://discover.nci.nih.gov/cimminer/). The lists of genes in each GO category are accessable in File S1.

### Parameters of instability

The several parameters of genomic instability used in the present manuscript (Figure 5B) have been described previously [37].

## Results

### Determination of relative kinetochore transcript expression profiles in the NCI-60

For this analysis, we chose 21 well-characterized kinetochore genes. Seventeen of them were form the CCAN complex within the inner kinetochore [12] [13]; four additional genes (CENPE, CENPF, MIS12 and INCENP) were chosen for their important roles in maintenance of the functional kinetochore during the mitotic cycle. The relative transcript expression levels for these 21 genes are presented as average z scores in Figure 1A, using data compiled from five microarray platforms (HG-U95, HG-U133, HG-U133 Plus 2.0, GH Exon 1.0 ST from Affymetrix, Inc., and the Whole Human Genome Oligo Microarray from Agilent Technologies, Inc.). Average z scores were determined for each gene using their probe sets (Affymetrix) and probes (Agilent) that passed quality control criteria (see Materials and Methods). Intensity values were then converted to z scores by subtracting the 60-cell mean, and dividing by the standard deviation.

The linear range of the average expression for these genes across the NCI-60 went from 3.4 fold for CENPO to 20.7 fold for CENPF (converted from the log_2_ values given in Figure 1A, second to last row). The mean log2 intensities had an average of 6.71, with a low of 4.67 for CENPQ, to a high of 8.15 for CENPF (Figure 1A, bottom row).

The average of the 21 z score values for each cell line (from Figure 1A) is presented in Figure 1B as a composite of the abundance of kinetochore transcripts in each cell line, with SF-268 having the highest and NCI-H226 the lowest composite levels.

### Identification of a coordinate transcript pattern for kinetochore genes in the NCI-60

The patterns of relative expression of the 21 kinetochore genes from Figure 1A are compared to one another using Pearson's correlation analysis in Figure 2A. The red-colored correlations are statistically significant at p<0.05 (without multiple comparisons correction). Of the 210 total correlations in Figure 2A, there were 97 (46%) that were positive, and 0 that were negative at statistically significant levels. The genes with the highest number of significant positive correlations to other kinetochore genes were CENPE and CENPW, with 15 and 13, respectively, followed by CENPC, CENPN, CENPP, CENPQ, CENPU, and MIS12 with 12 significant positive correlations (Figure 2A, second to last row). The genes with the lowest number of significant positive correlations to other kinetochore genes were CENPT, CENPB, CENPI, CENPK and CENPK with 4, 5, 6 and 6 significant positive correlations, respectively.

Figure 2B presents the Figure 1A expression data in cluster image map format. The image indicates an absence of strong internal patterns for the 21-gene set. The cluster branches on the x-axis also indicate a general lack of tissue-of–origin specificity. However, the side-by-side locations (on the x-axis) of the cell lines MDA-MB-435, its ERBB2-transfectant MDA-N, and the genotypically associated M14 [38] indicate some cell-based specificity of signature.

### Comparison of the relative kinetochore transcript patterns to that for all other genes

In order to control for array bias for the robust positive correlations demonstrated between the kinetochore genes in Figure 2A, the transcript expression level z scores for each of the 21 kinetochore genes were compared to the pool of transcript expression level z scores for all other available genes. For each kinetochore gene, 21 genes were selected at random from the available 16,820 gene pool 100,000 times and compared by correlation. Figure 3 displays the distribution of these correlations. A slight positive bias was found. Taken as a whole there were 12.2%, and 6.0% of genes that had statistically significant correlations (in the absence of multiple comparisons correction) at p≤0.05 that were either positive, or negative, respectively. However, this bias is insufficient to explain the robust pattern of positive correlations seen in Figure 2A, which when compared to the Figure 3 results are found to be statistically significant with p<1×10^−6^.

### Transcription factor analysis for the kinetochore genes identifies candidates for their regulation

In order to determine whether transcription factors might be influential in the observed coordinate regulation of kinetochore genes seen in Figure 2A, we reviewed 399 transcription regulators for potential binding sites to the known kinetochore genes (Figure 4A) using the ABCC GRID Promoter Feature Extraction Page (http://grid.abcc.ncifcrf.gov/promoters/promoterInfo.php). Data was available for 11 out of the 21 kinetochore genes. Based on the number of average transcription factor binding sites present per gene, the top 28 transcription factors were identified. These had a range of 22.6 to 1.7 transcription factor binding sites present per gene. The transcript expression levels z scores (calculated as in Figure 1A) of these transcription factors were next correlated to the 21 kinetochore genes. The average of each transcription factor's correlation (to the 21 kinetochore genes) was then compared to that of all 16,820 available genes, and the significance of enrichment (if any) calculated. Those transcription factors with i) greater than or equal to 1.7 recognized binding sites in the kinetochore genes (the first column of numbers in Figure 4A), ii) statistically significant correlation to individual kinetochore genes (p<0.05), and iii) statistically significant enrichment (p<0.01) of the number of binding sites (in the absence of multiple comparisons correction) as compared to all genes (the last column of numbers in Figure 4A) are presented in Figure 4A.

There were four transcriptional regulators that meet the above criteria, CREB1, E2F1, FOXE1, and FOXM1. These factors have significant correlation to 15, 14, 13, and 7 of the kinetochore genes, respectively. All kinetochore genes except CENPT had at least one transcriptional regulator that met the above criteria.

### Copy number of kinetochore genes in the NCI-60 cell lines

Because amplification of chromosomal regions is common in cancer cell lines, we determined DNA copy numbers for each of the 21 kinetochore genes, for each of the NCI-60 cell lines using our NimbleGen HG18 CGH WG Tiling v2.0 array, as described previously [33]. The range of the estimated DNA copy number differences (maximum minus minimum) across the NCI-60 for these genes were from 1.78 for CENPF to 4.14 for CENPM. The average copy number for these genes in the NCI-60 was 2.32. Significant correlations were found between DNA copy number and expression for nine kinetochore genes (Figure 4A, bottom row).

### Assessment of potential microRNA influence on expression of kinetochore genes

The expression levels of 365 microRNAs with detectable expression in at least 10% of the NCI-60 as measured using the Agilent Technologies Human miRNA Microarray (V2) [29] were correlated to the expression levels of the 21 kinetochore genes (Figure 1A). Those found to have significant correlation were checked for predicted pairing of target regions between the 3′ end of the kinetochore gene and the microRNA (as defined by http://www.targetscan.org/). Those gene/microRNA pairs found to pass both these criteria are presented in Table 1.

### Functional categorization of genes whose expression patterns are significantly correlated to those of the kinetochore genes

The 21 kinetochore genes expression patterns from Figure 1A were correlated to those of 16,820 available genes. The genes whose expression patterns were correlated at statistically significant levels (without multiple comparisons correction) were determined. These 21 gene lists were then compared to all available genes for the purpose of identifying functional categories that were enriched using High-Throughput GoMiner (http://discover.nci.nih.gov/gominer/htgm.jsp). There were 29 categories, as defined by the GO Consortium (http://www.geneontology.org/GO.downloads.ontology.shtml), with significant change (colored red) for at least 11 kinetochore genes (displayed in Figure 5A). The lists of genes significantly correlated to the kinetochore gene from each GO category are accessable in File S1.

Of these categories, the predominant themes were cell cycle, mitosis and cell division (including GO:0000075, 0000280, 0007067, 0000087, 0000279, 0007049, 0000278, 0022402, 0022403, 0006260, and 0006259). Also present were chromosomes or chromatids (GO:0007059, 0000818, 0000070, and 0051276), and cellular response to stimuli, stress or damage (GO:0051716, 0033554, 0006974, and 0034984). The genes with the highest number of significant correlations to these functional categories were CENPK, INCENP, CENPW, and CENPU. The genes with the least number of significant correlations to these functional categories were CENPB, followed by CENPI and CENPT.

### Association of kinetochore gene expression to genomic instability

The 21 kinetochore genes expression patterns were correlated to several parameters of karyotypic complexity [37]. The number of clonal structurally rearranged chromosomes (S), the numerical complexity (N), the index of numerical heterogeneity (INH), the fraction of normal chromosomes that experience numerical heterogeneity (NCNH), and the fraction of abnormal chromosomes that experience numerical heterogeneity (ACNH), had predominately negative significant correlations (20/24, presented in bold red type in Figure 5B) when compared to the expression of the (21) genes involved in kinetochore function. Each of these functional parameters of karyotypic complexity had significant negative correlations to at least three of the kinetochore gene expression patterns. Negative correlation suggests that as the expression of the kinetochore gene is reduced, the instability increases. Alternatively, genomic instability may effect expression of these kinetochore genes.

CENPK stands out as having significant negative correlation to all five instability parameters. Comparison between the z score averages and the modal chromosome numbers of the cell lines as was done as for the five instability parameters in Figure 5B (values not shown), but yielded a lack of significant correlations.

## Discussion

While there are approximately 90 genes that have been described as being involved in the kinetochore [5], [6], [13], we selected for the current study 21 that are well-characterized, and have been proposed to be essential for kinetochore assembly and maintenance. Of these, 17 form the CCAN complex within the inner kinetochore, a set of genes that are constitutive elements of the human kinetochore [12], and four play important roles in the maintenance of the functional kinetochore during the mitotic cycle [13], [16], [17], [18].

The relative kinetochore gene transcript expression levels of RNA purified under strictly controlled cell cultures, and using quality-controlled probes derived from five microarray platforms [29], [32] results in a high level of reliability for this analysis. The use of transcript z scores [36] facilitated this analysis, as it allows data comparison across multiple platforms, despite differences in means and/or standard deviations [33]. This allowed the inclusion of more total probe sets, increasing confidence levels due to the high levels of reproducibility found between them. Taken as an average, the percent of probe sets that passed the quality control criteria in Figure 1A (described in Materials and Methods) for the 21 kinetochore genes matched that for the 16,820 all gene set, at 47.8 and 47.8% respectively. Lower percentages were found for CENPP and CENPR, at 20 and 22%, respectively, suggestive of either reduced probe specificity or potential splice variation for these genes.

The identification of the large number of positive significant correlations (Figure 2A) between the kinetochore gene expression levels identifies for the first time a general co-regulation of these genes in the NCI-60 cell lines. To place that observation into context, the distribution of correlations for each of the kinetochore genes as compared to all other (16,820) available genes was determined (Figure 3), and found to approach normal, with slight bias to the positive side in some cases. Thus, for the first time we have identified a coherent pattern of expression for these 21 kinetochore genes across these 9 tissue-of-origin types of cancer.

We next proposed that these results may be explained by the presence of a multi-factorial regulatory mechanism. Two potential regulatory influences for these genes were reviewed that might apply to normal cells; transcription factors (Figure 4), and microRNAs (Table 1). Each of these was shown to have a potential influence on a portion of the genes. The most strongly correlated group among these two was the set of four transcriptional regulators CREB1, E2F1, FOXE1, and FOXM1, with 49 significant positive correlations to kinetochore gene expression (Figure 4A). The microRNAs were next, with 11 significant microRNA / gene pair correlations. Taken together, these two classes of potential regulators provide a range of from none to six prospective regulatory influences for each of the kinetochore genes, with an average of 2.86 (per kinetochore gene). These observations have added significance due to the surprising lack of literature on potential regulatory elements affecting the kinetochore genes. Recently it was shown that reduction of the level of HJURP encoding a CENPA-loading factor results in reduction of the CENPA levels at centromeres, and kinetochore disfunction. [39]. In the current study transcript levels of these two genes in the NCI-60 are found to have a significant positive correlation of 0.534, suggesting that the HJURP gene may be co-regulated with kinetochore genes. Due to the relative dearth of information, potential regulators identified in this study are candidates for the future experimental work. Although other mechanisms of regulation (such as those that affect translation and protein modification) are not addressed here, the transcription mechanism may be critical in the maintenance of a coordinated level of kinetochore gene products.

While it has been demonstrated that the kinetochore consists of a group of highly conserved, and interdependent proteins [40], specific interaction data between kinetochore proteins is limited [41], [42] and additional proteins may also be involved in kinetochore assembly and function [40]. The functional groups for genes found to be enriched by correlation to the expression levels of the kinetochore genes in Figure 5A are largely associated with known kinetochore functions. These include cell cycle, mitosis, nuclear division, chromatid segregation, and chromosome movement and segregation.

The association of mis-regulation of some kinetochore genes with increased karyotypic instability and copy number variations seen in Figure 5B is consistent with prior reports that imbalance in expression of these genes results in impairment of kinetochore assembly, mitotic defects and aneuploidy [15], [23], [39], [43], [44]. Over-expression of several kinetochore genes has also been reported in cancer tissues [20], [21], [22], [23], supporting the hypothesis that kinetochore-associated genes may in fact function as proto-oncogenes. Although the kinetochore genes correlate to one another in many instances in a positive and statistically significant manner as shown in Figure 2A, the patterns (across the NCI-60) are not identical, as would be indicated by correlation values of 1.00. This partial overlap leaves adequate room for variability in results when comparing the kinetochore gene expression patterns to other patterns, such as the genomic instability parameters in Figure 5B. Addition of comparably controlled non-cancerous materials might provide insight into the range of expression variability of these genes tolerated by cells prior to kinetochore dysfunction.

Gene expression profiles have been used recently in multiple capacities in the context of furthering the understanding of cancer at the molecular level. These include, but are not limited to, the affect of alteration of a single gene's expression on the function of a group of genes [45], [46], the diagnosis and sub-classification of disease types [47] , the response to radiation [48], the association of functional groups of genes with disease progression [49], and their use in predicting metastasis [50], [51]. In the current study, we extend that list by profiling a defined functional group of genes for the purpose of identifying co-regulation of those genes. To the best of our knowledge, this is the first time this has been done. We presume that the utility of this panel for such studies will be greatly increased when sequencing of all coding regions in NCI-60 cell lines is completed.

To summarize, we utilized the NCI-60 cell line panel to identify for the first time co-regulation of a group of 21 core kinetochore genes. We identified a putative multi-factorial form of their regulation, including transcription factors and microRNAs. We strengthened the association between the variability of the expression of genes involved in kinetochore function and karyotypic instability. More broadly, we demonstrated the usefulness of the NCI-60 for broadening the understanding of fundamental cellular processes, such as kinetochore function. We propose that the comparison of expression profiles in the NCI-60 cell line panel could be used for the identification of other gene groups, the products of which are involved in assembly of multi-protein complexes of organelles.

## Supporting Information

File S1 **Gene lists for the** **Figure 5A** **GO categories.** For each GO category those genes are listed with significant correlation to the kinetochore gene for that file. The genes in each GO category are organized as 21 Excel files, one for each kinetochore gene. Each of these gene files includes the 29 GO categories from Figure 5A in the order presented there. The GO categories that appear as red blocks in Figure 5A appear in red text in the Excel files. The GO categories that appear as blue blocks in Figure 5A appear in blue text in the Excel files.
(XLSX)
